# Structure and assembly of ESCRT-III helical Vps24 filaments

**DOI:** 10.1126/sciadv.aba4897

**Published:** 2020-08-19

**Authors:** Stefan T. Huber, Siavash Mostafavi, Simon A. Mortensen, Carsten Sachse

**Affiliations:** 1 European Molecular Biology Laboratory (EMBL), Structural and Computational Biology Unit, Meyerhofstraße 1, 69117 Heidelberg, Germany.; 2 Ernst-Ruska Centre for Microscopy and Spectroscopy with Electrons (ER-C-3/Structural Biology), Forschungszentrum Jülich, 52425 Jülich, Germany.; 3 JuStruct: Jülich Center for Structural Biology, Forschungszentrum Jülich, 52425 Jülich, Germany.; 4 European Molecular Biology Laboratory (EMBL) Hamburg c/o DESY, Notkestraße 85, 22607 Hamburg, Germany.; 5 Department of Chemistry, Heinrich Heine University, Universitätsstraße 1, 40225 Düsseldorf, Germany.

## Abstract

ESCRT-III proteins mediate a range of cellular membrane remodeling activities such as multivesicular body biogenesis, cytokinesis, and viral release. Critical to these processes is the assembly of ESCRT-III subunits into polymeric structures. In this study, we determined the cryo-EM structure of a helical assembly of *Saccharomyces cerevisiae* Vps24 at 3.2-Å resolution and found that Vps24 adopts an elongated open conformation. Vps24 forms a domain-swapped dimer extended into protofilaments that associate into a double-stranded apolar filament. We demonstrate that, upon binding negatively charged lipids, Vps24 homopolymer filaments undergo partial disassembly into shorter filament fragments and oligomers. Upon the addition of Vps24, Vps2, and Snf7, liposomes are deformed into neck and tubular structures by an ESCRT-III heteropolymer coat. The filamentous Vps24 homopolymer assembly structure and interaction studies reveal how Vps24 could introduce unique geometric properties to mixed-type ESCRT-III heteropolymers and contribute to the process of membrane scission events.

## Teaser

The cryo-EM structure of Vps24 homopolymer filaments reveals the structural basis for ESCRT-III lipid assemblies.

## INTRODUCTION

Multivesicular body (MVB) formation, cytokinesis, and viral budding are membrane deformation events that share a common topology by pushing membrane away from the cytosol. The ESCRT machinery is evolutionarily conserved across eukaryotes and consists of five complexes: ESCRT-0 (mediates clustering of ubiquitinated cargo), ESCRT-I, ESCRT-II (involved in membrane budding), ESCRT-III (membrane budding), and Vps4 (ESCRT-III dynamic disassembly including membrane constriction up to scission) (*1*). The complexes are believed to assemble in a sequential manner during MVB biogenesis (*2*). Budding yeast has a total of eight ESCRT-III proteins with the core subunits Vps20, Snf7, Vps24, and Vps2, as well as the accessory proteins Did2, Vps60, Chm7, and Ist1 (*3*). In humans, there are 12 ESCRT-III proteins called CHMP (charged multivesicular body proteins). ESCRT-III proteins have a high sequence and structural similarity, sharing a common four-helix bundle motif, as known from x-ray structures (*4*–*7*). ESCRT-III proteins are directly involved in membrane remodeling and scission. More recently, a near-atomic resolution cryo–electron microscopy (cryo-EM) structure of the assembled reverse topology ESCRT-III IST1-CHMP1B has become available (*8*), which is thought to be involved in the membrane scission events of the late endosome. In addition to the best characterized processes of MVB biogenesis, cytokinesis, and viral budding (*9*–*11*), the ESCRT machinery has been found to drive many other membrane remodeling processes such as nuclear envelope reformation, plasma membrane repair, lysosomal protein degradation, and autophagosome closure (*12*–*15*).

In analogy to other membrane deforming systems such as clathrin or dynamin in endocytosis, it is thought that ESCRT-III proteins require large defined assemblies to perform the budding and scission reaction at the membrane by forming hetero-oligomer or hetero-polymer structures (*16*). In vitro studies using purified proteins demonstrated that either Snf7 and Vps24 alone or mixtures of ESCRT-III components, such as CHMP2A/CHMP3 or IST1/CHMP1B, form a variety of large macromolecular assemblies. Observed structures include sheets, strings, rings, filaments, tubules, domes, coils, and spirals (*8*, *17*–*19*), as reviewed recently (*3*). Using quantitative fluorescence lattice light-sheet microscopy, the stoichiometry of *Saccharomyces cerevisiae* ESCRT-III members Snf7 and Vps24 was estimated at a ratio of ~4:1 to 5:1 (*20*). These structures can be disassembled in vitro by Vps4 by unfolding ESCRT-III units (*21*, *22*). Dynamic turnover driven by Vps4’s adenosine triphosphatase (ATPase) activity has also been shown to support growing ESCRT-III filaments (*23*). How ESCRT-III proteins together with Vps4 accomplish the process of membrane scission still remains to be established (*3*, *23*). On the basis of the observation of dome structures from ESCRT-III polymers (*19*), it is thought that tapering ESCRT-III structures together with Vps4’s disassembly activity could drive membrane constriction and, finally, scission (*24*). Another model is based on the observation of ring-like structures on membrane surfaces that buckle and thereby initiate inward vesicle budding (*16*, *25*).

Despite the wealth of structural information on the ESCRT proteins available on different resolution scales, a detailed description of the organization of a canonical ESCRT-III subunit assembly into the observed ultrastructures is lacking. Thus far, the EM structures of Vps24 filaments and CHMP2A/CHMP3 tubules have been determined at 25- and 22-Å resolution, respectively (*18*, *26*). The interpretation of the densities by atomic models has been hampered by the relatively low resolution of the reconstructions. The recently determined cryo-EM structure of an inverted IST1-CHMP1B ESCRT-III tubular assembly (*8*) is the first high-resolution structure of a polymeric assembly, but it remains to be established how more regular topology complexes are organized. Here, we present the 3.2-Å resolution cryo-EM structure of an extended Vps24 conformation organized in a double-stranded helical filament. Interaction with negatively charged lipids contributes to the disassembly of the filaments as they are solubilized into smaller oligomers. Upon further addition of ESCRT-III partners Vps2 and Snf7, liposomes yield neck and tubular structures including an ESCRT-III heteropolymer coat. The here-determined cryo-EM structure of filamentous homopolymers of Vps24 has unique assembly properties that could extend the flexible geometries of mixed-type ESCRT-III heteropolymers.

## RESULTS

### Cryo-EM structure of Vps24 filaments reveals an extended Vps24 conformation

**Fig. 1:**
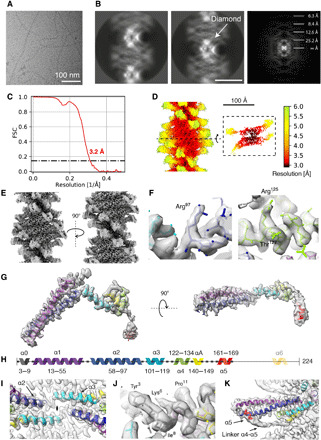
Cryo-EM structure of the ESCRT-III Vps24 filament. (**A**) Representative micrograph of ice-embedded Vps24 filaments. (**B**) Two 2D class averages of Vps24 and power spectrum derived from 2D class averages. (**C**) FSC curve indicating a resolution of 3.2 Å according to the 0.143 cutoff. (**D**) Local resolution mapped onto surface of cryo-EM map showing variation from 2.9 to 6.0 Å. (**E**) Two side views of Vps24 filament cryo-EM map related by a 180° rotation around the filament axis. (**F**) Typical side-chain density detail in the filament core. (**G**) Carved cryo-EM density of an extended Vps24 molecule. (**H**) Primary structure of Vps24 with color-coded seven helices used in the figures throughout the manuscript. (**I**) Connecting loop density between helix α2 and helix α3 viewed along the twofold symmetry axis (*d* axis). (**J**) Cryo-EM density of N-terminal helix α0 in the filament core. (**K**) Density of linker stretch connecting helix α4 and α5 at the outside of the filament.

To address the open structural questions on the molecular organization of canonical ESCRT-III assemblies, we purified and enriched full-length Vps24 filaments at high concentrations of 5.0 mg/ml (*18*) and observed plunge-frozen filamentous sample in vitreous ice by cryo-EM (Fig. 1A). Inspection of class averages revealed a 160-Å-wide filament with a diamond-shaped repeat pattern every 140 Å along the filament axis making up the half-pitch of the filament (Fig. 1B). The class average showed remarkable level of detail including secondary structure features. The corresponding Fourier spectrum revealed layer lines up to ~6 Å indicating the ordered helical organization of the filament. Using the class averages and a real-space approach (*27*), we determined the helical symmetry and found that 11.09 units per turn make up a pitch of 279 Å. From a total of 313,554 segments, we computed a three-dimensional (3D) reconstruction at an overall resolution of 3.2 Å (Fig. 1C and Table 1) with local resolution ranging from 2.9 Å in the filament core to 6.0 Å in the outer periphery (Fig. 1D). The outer density displays a series of long α-helical hairpins projecting outward from the filament center (Fig. 1E). The cryo-EM core density showed an expected level of main-chain and side-chain detail to allow building an atomic model of Vps24 (Fig. 1F). The initially placed four-helix bundle CHMP3 homology model [Protein Data Bank (PDB) ID 3FRT] (*4*) was largely covered by the density except for additional α-helical density corresponding to the N terminus, which was truncated in previous ESCRT-III models. After tracing the polypeptide chain residue by residue, however, it became clear that Vps24 is found in an extended conformation in the filament (Fig. 1G). In comparison with the compact four-helix bundle of human CHMP3 (PDB ID 3FRT), the continuous cryo-EM density loop (99 to 102) between helix α2 and helix α3 opens up so that Vps24 adopts an extended conformation (Fig. 1, H and I). Next to this loop, the cryo-EM density also contains a local twofold symmetry axis, which we term the dimer or *d* axis as it relates the packing of two opposing Vps24 molecules to a domain-swapped dimer. The N-terminal helix α0 including positive K5 and K6 points into the core of the filament (Fig. 1J). The final model connects helix α4 by a traceable linker stretch (150 to 160) up to helix α5 (161 to 169), which corresponds to a tubular density at the periphery and packs against the α1′-α2′ helix hairpin of the domain-swapped dimer (Fig. 1K). Together, although the C-terminal residues (170 to 224) could not be assigned due to the lack of density in the cryo-EM map, the Vps24 atomic model could be successfully built for a large part of the molecule (1 to 169) (fig. S1A).

**Table 1:** Cryo-EM data collection and model refinement statistics.

**Data collection and processing**	**Vps24**
Magnification	×130,000
Voltage (kV)	300
Electron exposure (e^−^/Å^2^)	40
Defocus range (μm)	0.75–3.0
Pixel size (Å)	1.040
Detector	Gatan K2 Summit
Symmetry imposed	C1 and helical
Final no. of particle images	313,554
Helical rise (Å)	25.18
Helical twist (°)	−32.47
Global map resolution (Å, FSC = 0.143)	3.2
Local map resolution range (Å)	2.9–6.0
**Model refinement**	**Vps24**
Initial model used (PDB code)	Homology model of PDB ID 3FRT*
Model resolution (Å, FSC = 0.143)	3.0
CC mask^†^	0.7972
Map sharpening B-factor (Å^2^)	−126
Model composition	
Nonhydrogen atoms	4 × 1381
Protein residues	4 × 170
RMSDs	
Bond lengths (Å)	0.008
Bond angles (°)	0.914
Validation	
MolProbity score	1.34
Clashscore^†^	3.65
EMRinger score^†^	1.02
Rotamer outliers (%)	0.64
Ramachandran plot	
Favored (%)	96.43
Allowed (%)	2.38
Disallowed (%)	1.19

*(4).

†Computed for a 20-mer.

After determining the Vps24 cryo-EM structure from the filament assembly, we compared it with the x-ray structure of human CHMP3 (PDB ID 3FRT) (*4*). On the basis of the two conformations, it is clear that the x-ray CHMP3 structure assumes a closed compact conformation, whereas the cryo-EM Vps24 structure is extended and presumably open (fig. S1B). The two conformations could be interconverted by a single movement at a hinge region between residues 99 and 102. This way, the two parts of CHMP3 can be structurally aligned with the extended conformation of Vps24. This analysis demonstrates that the polymer shows an open conformation as opposed to the closed conformation in the crystal structure. Further sequence comparison between Vps24 and CHMP3 reveals that Vps24 has a more positive N terminus when compared with CHMP3, where sequence identity is lowest (fig. S1C). When assembled, the Vps24 N terminus gives rise to a positively charged patch in the core of the filament.

To understand whether the here-determined filamentous cryo-EM structure of Vps24 can only be formed using purified proteins (~5 mg/ml) in solution, we tested lower concentrations of purified Vps24 from 0.5 to 4.0 mg/ml and incubated these samples for 1 or 24 hours. When inspecting negatively stained samples, we observed that filaments can form in all of these conditions (fig. S2). The low-concentration samples also contain a larger fraction of aggregated protein that did not assemble into filaments. The filaments formed at low concentration share the same twisted appearance and filament width as observed in high-concentration samples. The only notable difference is that high-concentration samples contain a higher number of filaments and fewer aggregates, which is helpful for cryo-EM structure determination. Together, these data confirm that the same type of Vps24 filaments can be formed at one order of magnitude lower protein concentration.

### Vps24 domain-swapped dimers form the basic unit of the ESCRT-III polymer

**Fig. 2:**
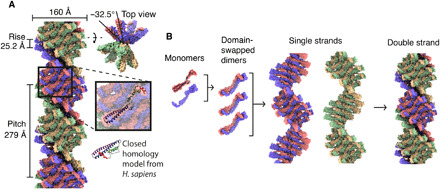
Assembly architecture of the double-stranded Vps24 filament. (**A**) Side and top views of the atomic model surface for the filament with labeled assembly parameters. The inset shows the position of a single Vps24 molecule. (**B**) Hierarchy of the assembly organization: monomer, the domain-swapped dimer, the single strand, and the resulting double-stranded filament.

The determined cryo-EM structure reveals the Vps24 conformation as well as the organization of Vps24 unit within the filament. Each extended Vps24 monomer packs against another Vps24 molecule to form a domain-swapped dimer. In this domain-swapped dimer, the helical repeating unit contains two Vps24 molecules that are distanced apart by 25.2 Å in translation and 32.5° by a left-handed rotation along the helical axis, making up a single protofilament (Fig. 2, A and B). Although the diamond feature of the class averages (Fig. 1B) suggested a dihedral symmetry with respect to the Vps24 filament axis, the high-resolution structure showed that the two helical protofilaments are not related by dihedral symmetry. In addition to the described *d* axis, which represents the domain-swapped dimer axis, there is a second local symmetry axis: the protofilament symmetry or *p* axis, as it relates two domain-swapped dimers by 170° rotation and thereby forms two opposing protofilaments (fig. S3, A to D). This *p*-axis symmetry deviates from 180° and therefore results in two distinct protofilaments including nonequivalent surfaces. Despite the observed difference in the overall protofilament structures, there is high conformational similarity between the dimers with root mean square deviation (RMSDs) of 0.66 Å for the main chain, after atomic coordinate refinement. Within the studied Vps24 filaments, the extended Vps24 conformation forms a domain-swapped dimer that makes up the basic unit of two distinct protofilaments.

### Critical intermolecular interactions are found within the protofilament of the double-stranded assembly

**Fig. 3:**
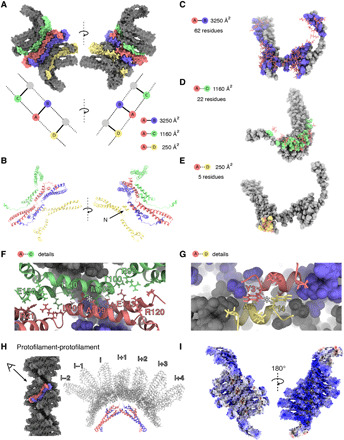
Molecular contacts in the Vps24 filaments. (**A**) Contacts between different Vps24 molecules within the filament. Space-filling representation of central blue-red dimer makes contacts to upper (green) and lower (yellow) neighbors. (**B**) One Vps24 molecule (red) with neighboring Vps24 molecules in ribbon presentation [color code as in (A)]. (**C**) Molecular residue contacts within a domain-swapped dimer. (**D**) Residue contacts between domain-swapped dimers and upper Vps24 neighbor. (**E**) Residue contacts between domain-swapped dimer and lower Vps24 neighbor. (**F**) Detailed view of the interface in (D) with an antiparallel alignment of residues M105-A109 centered by the asterisk. (**G**) Detailed view of contacting N termini shows antiparallel alignment of hydrophobic residues at asterisk. (**H**) Contacts between one domain-swapped dimer and seven dimers of the opposing strand are very scarce, resulting in a partial cavity. (**I**) Electrostatic potential map colored in units of k_B_T/e^−^ with red as −10 and blue as 10 shows the strong positive charge on the inner side of a single strand.

The extended monomer is stabilized by the domain-swapped counterpart as well as upper and lower neighboring subunits within the protofilament. The tightly integrated Vps24 molecule shares buried interaction surfaces with three neighboring Vps24 molecules inside a single strand of the assembly (Fig. 3, A and B). One monomer has 13,920 Å^2^ of total surface area, of which 3250 Å^2^ (62 residues) is engaged in interactions with the domain-swapped counterpart (Fig. 3C and fig. S3E), 1160 Å^2^ (22 residues) with the upper neighboring dimer (Fig. 3D and fig. S3F), and 250 Å^2^ (5 residues) with the lower neighboring dimer via the N termini (Fig. 3E and fig. S3G). In addition to the described *d* and *p* symmetry axes, there are local antiparallel arrangements of residues between the upper and lower neighboring subunits. Together with the upper monomer, they include hydrophobic pairs of (M105, A109) in the center of helix α3 as well as electrostatic interactions (R97, E150 and T100, E143) at the helix α3 ends and helix α4 (Fig. 3F). With the lower monomer, N-terminal helix α0 residues Y3, I4, and A7 stabilize long-reaching hydrophobic contacts within the filament core (Fig. 3G). The interactions between two protofilaments are scarce (N12, K133) and give rise to solvent-accessible cavities up to 10-Å distance between the molecules from the neighboring strands (Fig. 3H). When the two protofilaments are separated computationally, they show positively charged and hydrophobic surfaces that are not solvent exposed due to the architecture of the two protofilaments within the polymer (Fig. 3I). The majority of intermolecular interactions are, therefore, found within a protofilament, and the two protofilaments are joined by much fewer interactions.

### Lipid binding capacity of the Vps24 filament

**Fig. 4:**
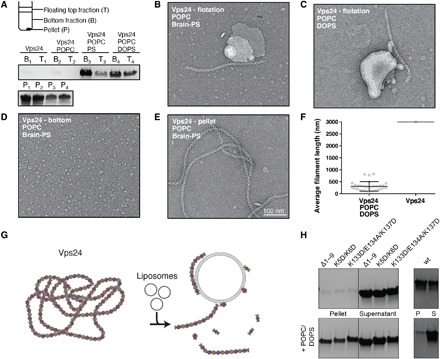
Vps24 filaments bind to negatively charged lipids and dissociate into shorter filament fragments. (**A**) Liposome flotation assays of Vps24 with POPC, POPC/PS, and POPC/DOPS lipid mixtures. In the presence of negatively charged lipids, Vps24 is found in the floating top fraction. Micrographs of negatively stained Vps24 of floating top fraction with (**B**) POPC/PS and (**C**) POPC/DOPS reveal filament fragments associated to dried liposomes. (**D**) Bottom fraction shows solubilized oligomer species. (**E**) Pellet fraction of Vps24 in the POPC/PS sample. (**F**) Quantification of filament lengths in the presence and absence of POPC/DOPS. When incubated with POPC/DOPS liposomes, Vps24 filaments shorten and associate with liposomes. (**G**) Depiction of filament disassembly by interaction with PS-containing liposomes. (**H**) Pelletation (top) and co-pelletation POPC/DOPS liposome assay of Vps24 mutants (left) (Δ1–9, K5D/K6D, and K133D/E134A/K137D) in comparison with wild-type (wt) Vps24 (right). Tested Vps24 mutants retain the ability to bind POPC/DOPS liposomes.

On the basis of the observation that the positive electrostatic and hydrophobic surfaces may be protected in the filament, we tested whether the Vps24 filament is capable of binding lipids using a liposome flotation assay. When concentrated to 3.0 mg/ml and incubated with negatively charged liposome preparations of POPC (1-palmitoyl-2-oleoyl-*sn*-glycero-3-phosphocholine)/DOPS (1,2-dioleoyl-*sn*-glycero-3-phospho-l-serine) and POPC/Brain-PS (1,2-diacyl-*sn*-glycero-3-phospho-l-serine) mixtures, Vps24 filaments are solubilized and partition to the floating liposome top fraction, whereas with POPC alone they pellet entirely as expected from the control (Fig. 4A). We inspected micrographs of negatively stained samples from the floating top fraction, and the images confirm that short Vps24 filaments are found associated with the liposomes (Fig. 4, B and C). Solubilized Vps24 oligomers are visualized from the bottom fraction (Fig. 4D) and long Vps24 filaments in the pellet fraction in the absence of any liposomes (Fig. 4E and fig. S4A). By measuring the length of the filaments, this qualitative observation is confirmed when comparing the liposome-bound floating top fraction with the pellet fraction (Fig. 4F). The lipid binding assays demonstrate that Vps24 has specific binding property to negatively charged lipids and that lipid binding solubilizes the filaments resulting in shorter filaments and oligomeric Vps24 species (Fig. 4G).

In our resolved Vps24 structure, we find helix α0 buried within the core of the filaments and show that lipid binding solubilizes filaments. Previously, helix α0 of Snf7 has been found to bind membranes (*28*). To clarify Vps24’s helix α0 role in lipid binding, we tested a series of Vps24 mutants with respect to filament formation and lipid binding capacity. First, we investigated mutations in helix α0, such as the truncation of nine N-terminal residues Vps24 (10 to 224) or the K5D/K6D mutant, and second, we analyzed the K133D/E134A/K137D charge-reversal triple mutant with mutations that lie outside of helix α0 but involved in critical intermolecular contacts within the filament. As expected from the design, both types of mutations abrogated filament formation confirmed by the absence of filaments in the electron microscope (fig. S4B). In support, when these mutants were subjected to a pelletation assay, we observed that the Vps24 mutants remain largely soluble in contrast to wild-type Vps24 (Fig. 4H). Upon addition of POPC/DOPS liposomes, however, the three Vps24 mutants co-pelleted with the liposomes, indicating direct lipid binding. Together, in the absence of the positive N-terminal helix α0 as well as a charge-reversal mutant, other stretches of the proteins can still bind to liposomes. The results of the liposome co-pelletation assays of the filament-breaking Vps24 mutants extend the candidate residues responsible for lipid binding beyond the N terminus.

### Vps24 interaction with ESCRT-III family members Vps2 and Snf7

**Fig. 5:**
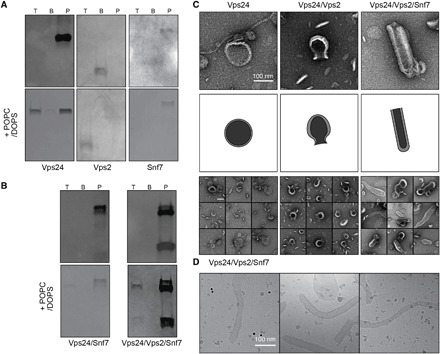
Vps24 interactions including Vps2 and/or Snf7 with POPC/DOPS liposomes at high and low concentrations. (**A**) Flotation (top) and POPC/DOPS liposome flotation (bottom) assays of Vps24, Vps2, and Snf7 alone. At high concentrations (3.0 mg/ml), Vps24 and Vps2 are found to bind liposomes. (**B**) Flotation (top) and liposome flotation (bottom) assays of Vps24/Snf7 and Vps24/Vps2/Snf7 mixtures. In binary and tertiary high-concentration ESCRT-III complexes, mainly Vps24 and Vps2 can be found to bind liposomes. (**C**) Negative-stain electron micrographs (top), sketched lipid outlines (middle), and galleries (bottom) of low-concentration Vps24, Vps2/Vps24, and Vps2/Vps24/Snf7 mixtures (0.5 mg/ml) incubated with POPC/DOPS liposomes. Scale bar, 100 nm. Left: Vps24 filaments can be found associated with intact spherical liposomes. Middle: When incubated with Vps2/Vps24, many liposomes have constricting neck structures. Right: The presence of Vps2/Vsp24/Snf7 reveals many tubular membrane structures. (**D**) Electron cryo-micrographs of low-concentration Vps2/Vps24/Snf7 mixtures confirm the presence of a striated protein coat on POPC/DOPS membrane tubules.

To further assess whether Vps24’s interaction with POPC/DOPS liposomes is affected by the presence of ESCRT-III partner proteins at high concentrations of 3.0 mg/ml, we performed the Vps24 flotation assay with Vps2 and Snf7 in the presence and absence of liposomes. First, we tested the interaction of the ESCRT-III proteins alone with liposomes, i.e., Vps24, Snf7, and Vps2 (10 to 166). Both Vps24 and Vps2 (10 to 166) migrate to the top floating fraction due to binding to the liposomes, whereas Snf7 does not appear to bind to the POPC/DOPS lipids and remains in the pellet fraction (Fig. 5A). Next, we tested the binary and tertiary mixtures consisting of equimolar ratios of Vps24, Snf7, and/or Vps2 and found that both Vps24 and Vps2 can be found in the top floating membrane fraction, whereas Snf7 remains in the pellet (Fig. 5B). Although Snf7 has been reported to bind to lipids (*28*), it is possible that the high concentration that was required here for band detection in SDS–polyacrylamide gel electrophoresis (PAGE) caused the formation of large Snf7 structures, an effect that has been reported previously (*29*), and the resulting Snf7 polymers are too heavy to float to the top fraction. Together, Vps24 and Vps2 are able to bind negatively charged POPC/DOPS lipids either by themselves or in binary or tertiary ESCRT-III mixtures with Snf7.

On the basis of the high-concentration interaction studies of Vps24 with ESCRT-III partners by liposome flotation, we set out to study the interaction of Vps24, Vps2, and Snf7 and their binary and tertiary combinations with liposomes at low protein concentrations of 0.5 mg/ml followed by negative-staining EM observations. After 24 hours of incubation, we found that the ESCRT-III proteins alone did not alter the shape of the liposomes (Fig. 5C). The Vps2/Vps24 binary mixture causes a neck formation at the liposome presumably by membrane constriction. Furthermore, the tertiary complex causes liposomes to take up tube or extended oval shapes. The binary and tertiary complex mixtures did not show any filaments compatible with the characterized Vps24 homopolymers. To verify that these membrane shape changes are induced by protein binding, we plunge-froze the tertiary mixture, observed it by cryo-EM, and found a striated protein coat pattern along the tube (Fig. 5D). On the basis of the results of low-concentration incubations of ESCRT-III with liposomes, Vps24 and Vps2 cooperate to induce initial neck formation on liposomes, whereas the further presence of Snf7 leads to an extension of these structures into elongated tubes.

## DISCUSSION

To shed light on the structure and organization of ESCRT-III assemblies, we determined the cryo-EM structure of Vps24 filaments. The structure reveals an extended Vps24 conformation tightly engaged in a domain-swapped dimer. These dimers extend to two distinct protofilaments with complementary charged and hydrophobic surfaces forming the filament. Furthermore, we demonstrated that the observed Vps24 polymer is capable of interacting with negatively charged lipids that induce dissociation into shorter and oligomeric Vps24 species.

The determined Vps24 structure in the filament corresponds to an elongated conformation extending to 100 Å in length. In contrast, previously determined x-ray structures of ESCRT-III proteins show a compact conformation with 70 Å in length (*4*, *5*) and have often been referred to as a closed state. Experimental activation of this closed conformation could be accomplished by C-terminal truncations (*30*), by disruption of helix α5 binding by R52E point mutant of Snf7 (*16*), or by increasing salt concentration (*31*). In our cryo-EM structure, we find that helix α5 binds to the hairpin formed by helix α1′/α2′ of the corresponding domain-swapped dimer. Although we determined our structure at 100 mM NaCl concentration, no activating displacement of helix α5 was required for extending the conformation in the polymer. Analogous observations have been made in another recently determined ESCRT-III assembly (*8*, *32*). In the respective IST1-CHMP1B tube, IST1 forms the outside coat of the assembly and is found in an extended conformation. Although it is not clear whether the polymer assembly represents an active form, our Vps24 domain-swapped dimer structure shows that extended conformation and binding of helix α1/α2 hairpin to helix α5 can coexist in a polymerized ESCRT-III assembly.

In addition to the first structural analysis of the Vps24 filament, in previous studies, many mutants were tested with respect to filament formation (*18*). The here-determined atomic model of Vps24 filaments now rationalizes two major classes of mutations that abrogate filament formation: (i) mutations in helix α3/α4 such as K133D/E134A/K137D and (ii) mutations in helix α0 such as K5/K6 as well as truncations of the N terminus (Δ1–9), both of which disrupt critical stabilizing residues of the domain-swapped dimer with lower and upper neighboring units (Fig. 3, F and G, and fig. S4B). Although in related ESCRT-III proteins helix α0 has been attributed to function as a membrane anchor and thereby revealing a competing binding between lipid and polymer interaction (*28*), we observe that helix α0 is also critical for generating the polymer core of the Vps24 filaments. To characterize the lipid binding capacity of Vps24 filaments, we performed liposome binding assays (Fig. 4). Supported by the determined cryo-EM structure, the ESCRT-III filaments preferentially bind negatively charged lipids presumably by a positive surface patch located at the protofilament interface. Furthermore, this interaction weakens the filamentous assembly and leads to dissociation into smaller filament fragments and oligomers of Vps24. This observation was not expected and adds a previously unknown aspect to the assembly/disassembly cycles of ESCRT-III polymers/oligomers. Thus far, Vps4 has been attributed as an adenosine triphosphate (ATP)–consuming catalyst of ESCRT-III assembly/disassembly, putatively acting as an active driver of membrane remodeling leading to constriction (*24*). Other ESCRT-III homopolymers such as Snf7 do not show this lipid-induced disassembly behavior. It remains open whether the here-described lipid-induced dissociation property of Vps24 will act in concert with the described AAA-ATPase unfolding activity of Vps4.

The here-determined Vps24 structure reveals the location of the N-terminal helix α0 in the core stabilizing the filament. In addition, we demonstrated that Vps24 filaments become solubilized and shortened by membrane interaction. This observation appears to contradict previous findings that suggested the N terminus of Snf7 to be solely responsible for in vitro lipid binding while being present in functional polymeric ESCRT-III assemblies (*28*). Similarly, CHMP2A’s N terminus was shown to be required for functional plasma membrane deformation (*17*). As helix α0 truncations of Vps24 still show binding to negatively charged liposomes, Vps24’s precise molecular binding properties may differ from those of Snf7 and CHMP2A. Furthermore, our experiments of Vps24 mixing with ESCRT-III partners and lipids show that major aspects of existing ESCRT models can be recapitulated: The Vps2/Vps24 binary mixture shows an enriched fraction of elongated liposomes including necks, and the Vps2/Vps24/Snf7 mixture displays many membrane tubules including a putative ESCRT-III protein coat in the absence of any filaments resembling the Vps24 homopolymers (Fig. 5). These observations are in line with data of human CHMP2A/CHMP3 assemblies that require lipid membranes to assemble into polymeric membrane coats (*26*). The here-determined cryo-EM structure of Vps24 homopolymers is morphologically very different in comparison with the binary and tertiary ESCRT-III complexes. Similarly, we expect that the biochemical properties can also change once Vps24 is engaged in complex ESCRT-III structures. In addition, our data suggest that although ESCRT-III subunits are closely related in sequence, their biochemical interaction data may not be generally transferable to the properties of other members of the ESCRT-III family. Consistent with our experiments, the observed Vps24 homopolymer solubilization upon lipid binding could provide a critical step for recruiting Vps24 into other hetero–ESCRT-III complexes and promoting the active membrane-deforming assembly.

Recent characterization using quantitative fluorescence lattice light-sheet microscopy at MVB scission sites (*20*) indicates that ESCRT-III structures consist of heteropolymers, suggesting an approximately fivefold excess of Snf7 over Vps24 molecules. There are three principal architectural aspects of the determined Vps24 filament structure, which have critical consequences for considering shape and geometry modulation of mixed-type hetero ESCRT-III assemblies: (i) apolar assembly, (ii) twisting properties, and (iii) double protofilament architecture. First, one important consequence of the determined cryo-EM Vps24 filament is that, despite its lack of exact dihedral symmetry, the filament is apolar with two adjacent protofilaments (Fig. 2) and, consequently, the assembly does not contain unique ends. This is in contrast to the previously proposed linear Snf7 filaments (*6*), which confer directionality by two unique interaction surfaces at the ends of the filament. Structural integration of Vps24 into linear Snf7 polymers can neutralize the polarity in such a way that two linear Snf7 polymers with opposite polarity could be combined (fig. S5A). Second, in contrast to the previously proposed Snf7 polymer models (*6*) and observed planar ring structures (*16*), the here-determined cryo-EM Vps24 filament has an intrinsic twisting propensity due to the helical symmetry. When interspersed in mixed polymers at the observed ratios, Vps24 will induce curvature to the otherwise flat Snf7 filaments (fig. S5B). These claims are corroborated by previous experiments that addition of Vps24 to planar Snf7 polymers deflects them into a third dimension (*6*, *16*). Third, the determined Vps24 filament is made of two protofilaments, which opens up the possibility of spatially combining multiple linear Snf7 polymers. In support, a recent study found that Vps2 and Vps24 polymerize side by side with Snf7 to form larger filament bundles (*23*), thereby supporting the notion that Vps24 can act as an adaptor by connecting multiple polar and apolar ESCRT-III homopolymers (fig. S5, C and D). Recent findings of yeast ESCRT-III with lipids from cryo–electron tomography support the discussed geometry arguments (*33*) but still lack the resolution to unambiguously fit different ESCRT-III molecules within the structure. Further theoretical arguments have been put forward that membrane remodeling may be driven by inclusion of monomers that cause different geometries, e.g., in a sequential polymerization model or a dynamic exchange model (*23*). Although our atomic model cannot favor one ESCRT membrane scission model over another, the here-determined cryo-EM homopolymer Vps24 structure reveals the structural basis for a geometrical adaptor and modulator function in ESCRT-III heteropolymers. In the future, it will be critical to elucidate the detailed structures of Snf7 and Vps24 complexes to better understand the geometries in mixed-type ESCRT-III polymers.

## MATERIALS AND METHODS

### Expression and purification of Vps24, Vps2, or Snf7 and Vps24 filament formation

Full-length Vps24 with a tobacco etch virus (TEV)–cleavable His-tag was expressed in BL21 RIPL cells using autoinduction medium. Cells were lysed by sonication, and the debris was cleared by ultracentrifugation. The supernatant was applied to an affinity 5-ml HisTrap HP column (GE Life Sciences, Marlborough, MA), and Vps24 was eluted using a 25-ml gradient from 0 to 300 mM imidazole. After removing imidazole by dialysis, the His-tag was removed by in-house produced TEV and separated using a second HisTrap column. Until this step, NaCl concentration was kept at 300 mM to reduce oligomerization. In the final purification step, gel filtration on a Superdex 200 16/600 column was performed exchanging the final buffer [100 mM NaCl, 20 mM tris (pH 8)]. After concentrating the pooled fractions on a Vivaspin 20-ml column with a 10-kDa cutoff to a final concentration of 0.24 mg/ml, aliquots were snap-frozen in liquid nitrogen. To induce filament formation, purified Vps24 was concentrated to 5.0 mg/ml over the Amicon Ultra-0.5 mL Centrifugal Filter with 3-kDa cutoff. The sample was incubated at 4°C overnight for complete polymerization. Vps24 mutants were purified in the same manner. Vps2 (10 to 166) was expressed as an N-terminal His-MBP-TEV construct and purified using the same protocol as Vps24. Full-length Snf7 was expressed with an N-terminal His-TEV tag and purified using nickel affinity chromatography followed by size exclusion chromatography as described above.

### Electron microscopy

To verify filament formation, we used negative-staining electron microscopy. Vps24 polymers were diluted by a factor of 10 and 100 and applied to continuous carbon grids that were glow-discharged in a PELCO easiGlow Glow Discharger (Ted Pella Inc., Redding, CA). Sample was blotted and stained with 2% uranyl acetate using the droplet technique. The grid was dried for 5 min on air and then imaged in a ThermoFisher Scientific/FEI Morgagni electron microscope. For electron cryo-microscopy, an additional ultracentrifugation step at 80,000 rpm (279,000*g*) in a TLA-100 rotor was done to enrich for polymers in the pellet. The 3.6-μl sample was applied to glow-discharged Quantifoil R2/1 grids and rapidly plunge-frozen in a propane/ethane (67:37) mix using a ThermoFisher Scientific Vitrobot Mark IV, set at 100% humidity and 10°C temperature. A total of 3257 micrographs were recorded using a ThermoFisher Scientific Titan Krios at 300 kV and Gatan K2 Bioquantum in counting mode operated by SerialEM (*34*). The 40-frame movies were imaged at an underfocus between 0.75 and 3.0 μm at a cumulative dose of 40 e^−^/Å^2^ and a pixel size of 1.04 Å per pixel.

### Image processing and helical reconstruction

MotionCor2 (*35*) was used to correct for stage-drift and beam-induced motion over the acquired frames. Last, a total of 1320 micrographs were selected based on specimen coverage, ice quality, and minimal drift. Selected micrographs were imported into RELION 2.1 (*36*), and contrast-transfer function (CTF) determination using Gctf (*37*) was performed. Filaments from a few micrographs were manually traced, extracted, and 2D-classified into 10 classes. Three of those were chosen as references for automated tracing in RELION, resulting in 524,155 coordinates with an extraction step of 24.7 Å. Segments with a box size of 256 pixels were excised at these coordinates with binning set to 2. Segments were subjected to 2D classification in RELION with 100 classes, a mask diameter of 260 Å, and 20 iterations. The data were cleaned at this stage, selecting 62 of 100 classes, corresponding to 313,554 of 524,155 (59.8%) segments. The remaining particles were reextracted and centered according to their position in the 2D classes. Initially, a smaller subset of dataset was used for symmetry determination. Class averages from RELION were rotated upright by 90° following the convention in SPRING (*27*), and the program “segclassreconstruct” from SPRING was used to identify candidates of helical symmetry parameters. Three candidate symmetries were identified at ~9.0, 11.0, and 13.0 units per turn with a pitch of ~275 Å. Only the symmetry solution with 11.0 units per turn converged to a solution with visible secondary structure features. Last, the complete segment stack was subjected to 3D autorefinement, allowing local searches in helical symmetry between −33° and −32° rotation and 24 to 25.5 Å for the rise corresponding to SPRING’s solution for helical symmetry. Last, the refinement converged to 25.18 Å for the rise and −32.47° for the rotation. The resulting 3D volume was used to create a mask for the central 30% of the density along the helical axis focusing on the central region to account for flexibility of Vps24 filaments, and refinement was restarted with the mask. Gctf was used to refine the CTF per particle, and the CTF parameters were updated in the star file from the final 3D reconstruction. Fourier shell correlation (FSC) curves were calculated within the same mask containing the central 30% of the volume along the helical axis. Postprocessing of the resulting densities was performed in RELION to automatically sharpen the maps using a B-factor of −124 Å^2^. Helical symmetry was imposed in real space onto the final 3D volume. Local resolution was assessed in RELION and rendered in UCSF Chimera (*38*).

### Cryo-EM map interpretation and model building

A homology model based on the human Vps24 protein CHMP3 with the PDB model 3FRT was created using the SWISS-MODEL (*39*) server and rigid body fitted by Chimera. Helix α5 of this model had to be slightly adjusted, and the eight N-terminal residues had to be manually added using Coot (*40*). Helices α3 and α4 diverged considerably from the homology model and were manually rebuilt. Several rounds of real-space refinement with Phenix (*41*) and manual inspection and optimization in Coot were performed. Three copies of the molecule were added to build a complete helical repeating unit, and the resulting 4-mer was expanded by helical symmetry twice in each direction, resulting in a total of 20 monomers present for the refinement. Models related by helical symmetry were held together by non-crystallographic symmetry (NCS) restraints, and another round of real-space refinement was performed. The symmetry-related models were removed, and the 4-mer comprising the helical repeating unit was saved as the final model. Electrostatic potentials were calculated using the web service of pdb2pqr (*42*) and the APBS software (*43*) with default settings. The resulting electrostatic maps were projected on solvent-excluded surfaces of the respective atomic models using ChimeraX (*38*). The potential was color-coded in units of kT/e^−^ with −10 as red and 10 as blue. Analyses of intermolecular contacts were done in ChimeraX using the “Find Contacts” and “Interfaces” functionality with standard settings. Local symmetry axes were found in Chimera by overlaying symmetry-related structures using the “MatchMaker” tool and the “measure rotation” command. Structure renderings were done in ChimeraX. For visualization of the weak-density loop between helices α4 and α5 at the same surface level as the rest of the structure, the density was sharpened using a model-based approach with the LocScale software (*44*).

### Liposome flotation, liposome pelletation, and low-concentration liposome incubation assays

Liposome flotation assays were performed in 20 mM tris (pH 8), 100 mM NaCl. Liposomes were formed using a mini-extruder with a 0.2-μm membrane. The following compositions were tested for binding Vps24: first, 100% POPC, second, 75% Brain-PS:25% POPC, and third, 75% DOPS:25% POPC. Vps24 and liposomes were mixed and incubated for 1 hour at 4°C. For the ESCRT-III complexes, Vps24, Vps2, and/or Snf7 were mixed at 0.1 mg/ml per protein and concentrated to 3.0 mg/ml before being centrifuged at 174,000*g* at 4°C for 1 hour. The pellet was resuspended and used for the liposome flotation assay. For Vps2, the sample was concentrated to 3.0 mg/ml and directly used in the flotation assay without centrifugation as the protein remained soluble. The sucrose centrifugation step was carried out at 174,000*g* for 1 hour at 20°C, and fractions were immediately collected for either negative-stain EM or SDS-PAGE analysis using InstantBlue (Expedeon) staining. For the liposome pelletation, purified proteins at 2 mg/ml were mixed with a 1:2 molar ratio of POPC/DOPS liposomes and incubated for 1 hour at 20°C. The mixture was then centrifuged at 175,000*g* for 45 min at 20°C, and the supernatant and the resuspended pellet were analyzed by SDS-PAGE. For the low-concentration liposome incubation assays, the proteins were concentrated to 0.5 mg/ml per protein, subsequently mixed with liposomes, and incubated overnight at 4°C before being examined by negative staining. To confirm that the N-terminal His-tag on Snf7 did not alter its behavior, both the flotation and low-concentration experiments with samples containing Snf7 were also performed while adding 1:2 molar ratio of TEV protease to the sample during the overnight or 1-hour incubations. The results remained the same. Lipids were bought from Avanti lipids or Merck.

## Supplementary Material

aba4897_SM.pdf

## SUPPLEMENTARY MATERIALS

Supplementary material for this article is available at http://advances.sciencemag.org/cgi/content/full/6/34/eaba4897/DC1

## Appendix

View/request a protocol for this paper from *Bio-protocol*.
