# Supplementary material for: Structure and assembly of ESCRT-III helical Vps24 filaments
Source: Sci Adv. 2020 Aug 19;6(34):eaba4897. doi: 10.1126/sciadv.aba4897 (PMC7438092; doi:10.1126/sciadv.aba4897)
Supplement: aba4897_SM.pdf [file aba4897_SM.pdf]

Science Advances
AAAS

advances.sciencemag.org/cgi/content/full/6/34/eaba4897/DC1

Supplementary Materials for

Structure and assembly of ESCRT-III helical Vps24 filaments

Stefan T. Huber, Siavash Mostafavi, Simon A. Mortensen, Carsten Sachse*

*Corresponding author. Email: c.sachse@fz-juelich.de

Published 19 August 2020, *Sci. Adv.* **6**, eaba4897 (2020)
DOI: 10.1126/sciadv.aba4897

This PDF file includes:

Figs. S1 to S5

Supplementary Figures

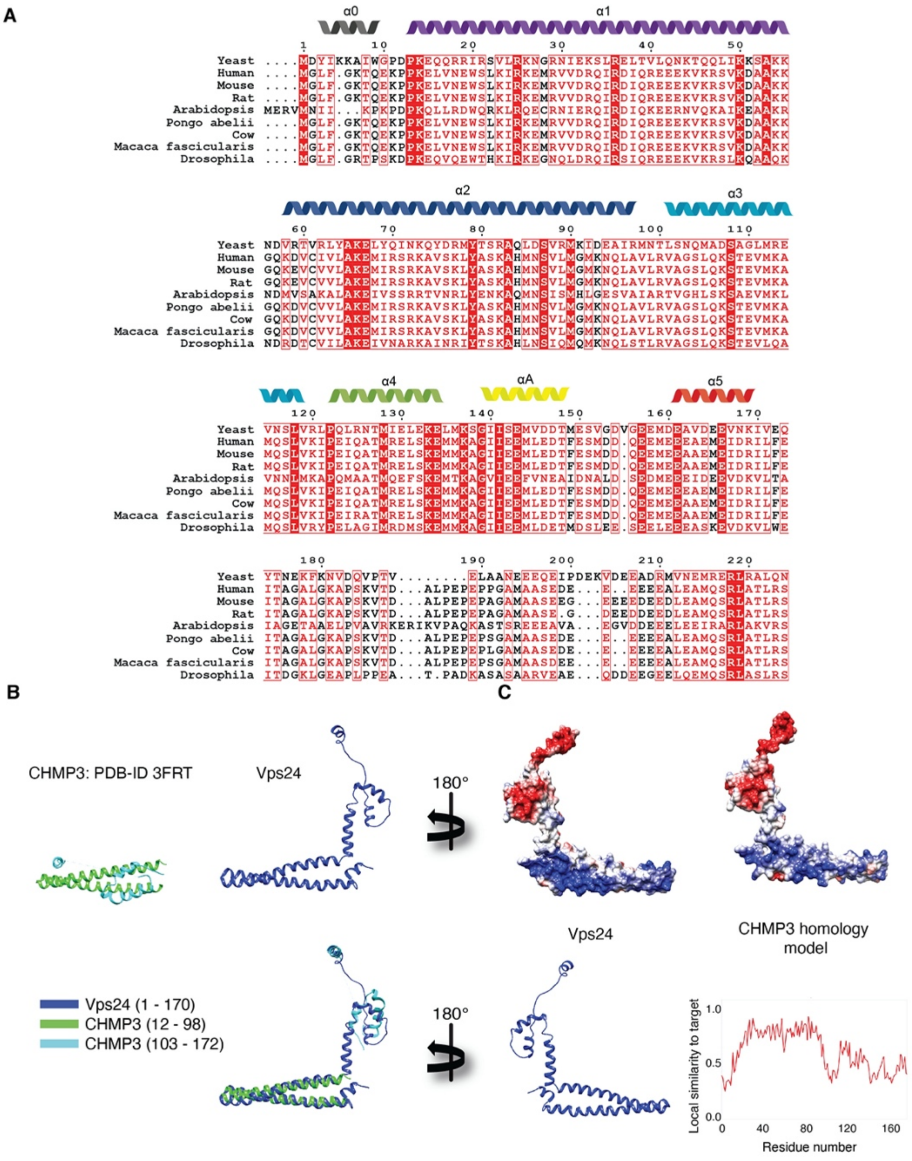

Figure S1: Multiple sequence analysis of related Vps24/CHMP3 sequences and structure comparison of CHMP3 and Vps24.

(A) Predicted secondary structures based on a Vps24 homology model are shown as pictograms. (B) Structure comparison of CHMP3 (PDB ID 3FRT) with cryo-EM Vps24 structure. CHMP3 is present in a closed whereas Vps24 found in an open conformation. The structures can be interconverted by a single movement at hinge residues 99 – 102. (C) Comparison of extended conformations Vps24 and corresponding CHMP3 homology model reveals the more pronounced N-terminal patch of positive residues in Vps24. According to a local similarity plot (bottom right), the sequence identity between the two ESCRT-III proteins is lowest at the N terminus.

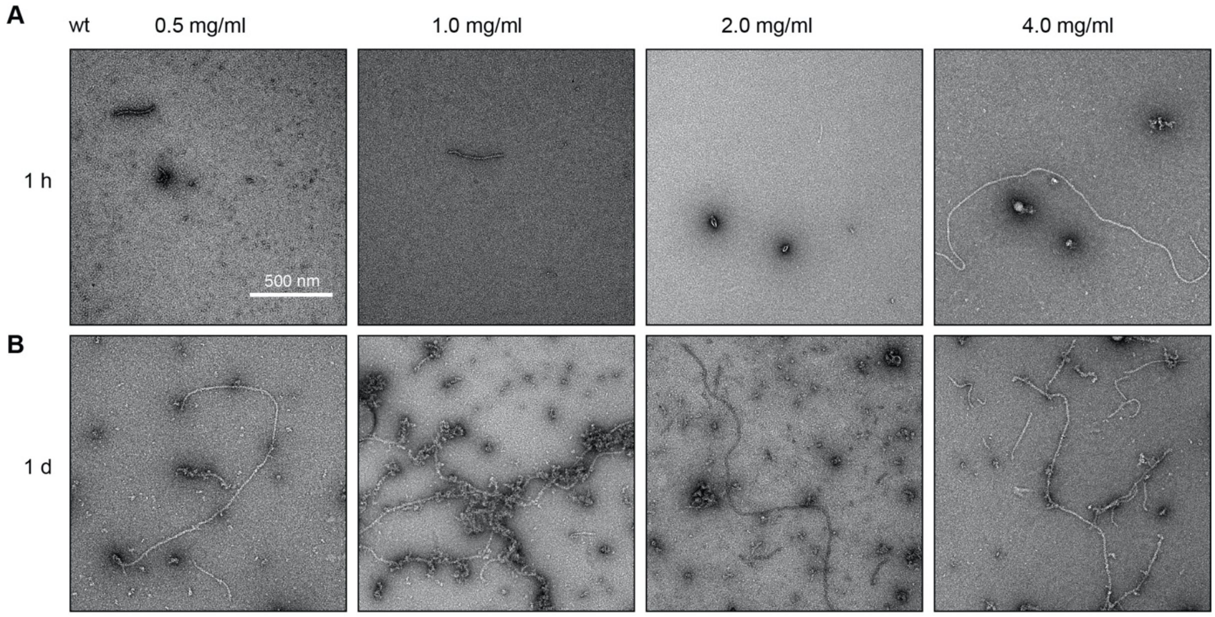

Figure S2: Vps24 filaments from low-concentration incubations.

Negative stain electron micrograph comparison of a concentration series of incubated Vps24 at 0.5, 1.0, 2.0 and 4.0 mg/ml after (A) 1 hour (top) and (B) 1 day (bottom) confirm the presence of filaments at low concentration.

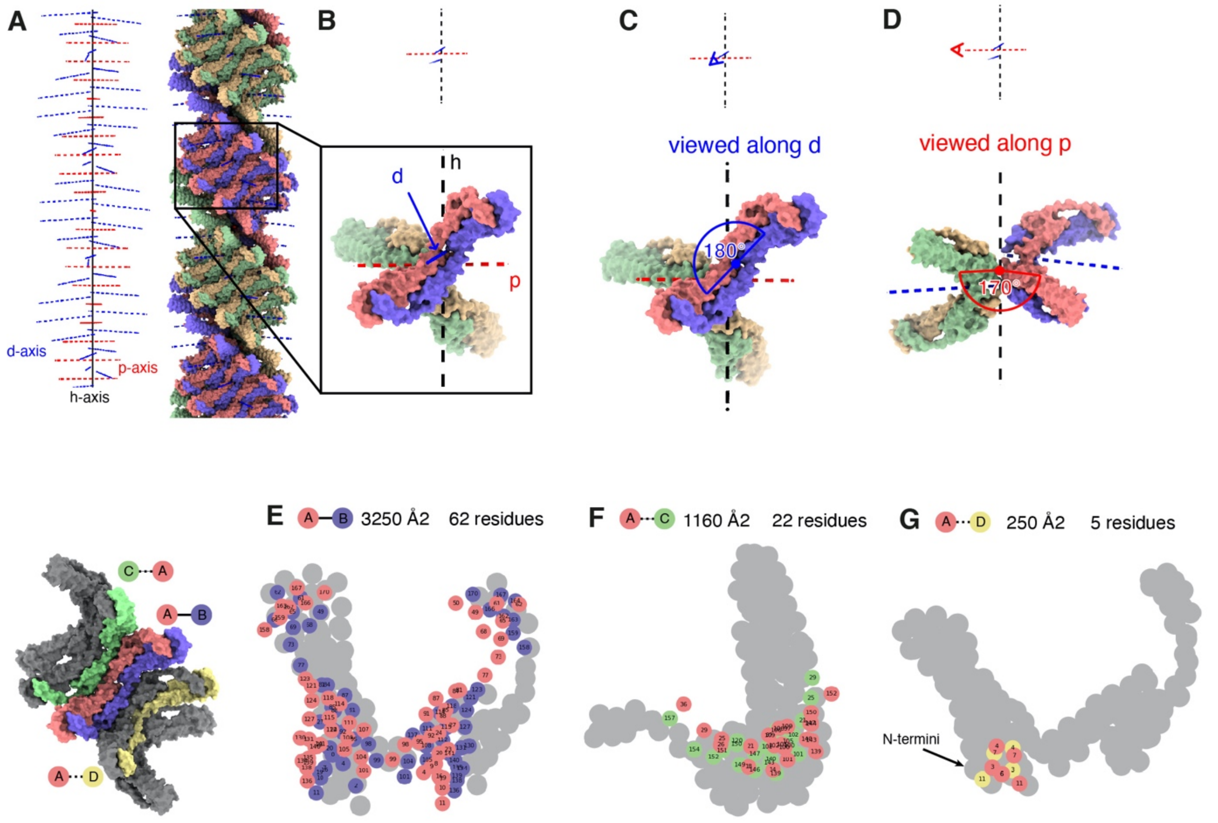

Figure S3: Vps24 filaments have two symmetry axes in addition to the helical symmetry axis and detailed molecular contacts in the Vps24 filaments.

(A) Vps24 filament with helical axis in black, dimer (*d*)-axis in blue and protofilament (*p*)-axis in red. (B) Inset of a helical repeating unit consisting of two dimers including respective symmetry axes. (C) View along the *d*-axis shows two Vps24 molecules (red, blue surface) related by a 180° rotation forming a domain-swapped dimer. (D) View along the *p*-axis shows that two domain-swapped dimers (green/yellow and blue/red surfaces) are related by a rotation of 170°. (E-G) Detailed projection of Figure 3 C, D, E with residue numbers highlighted that are involved in molecular contacts.

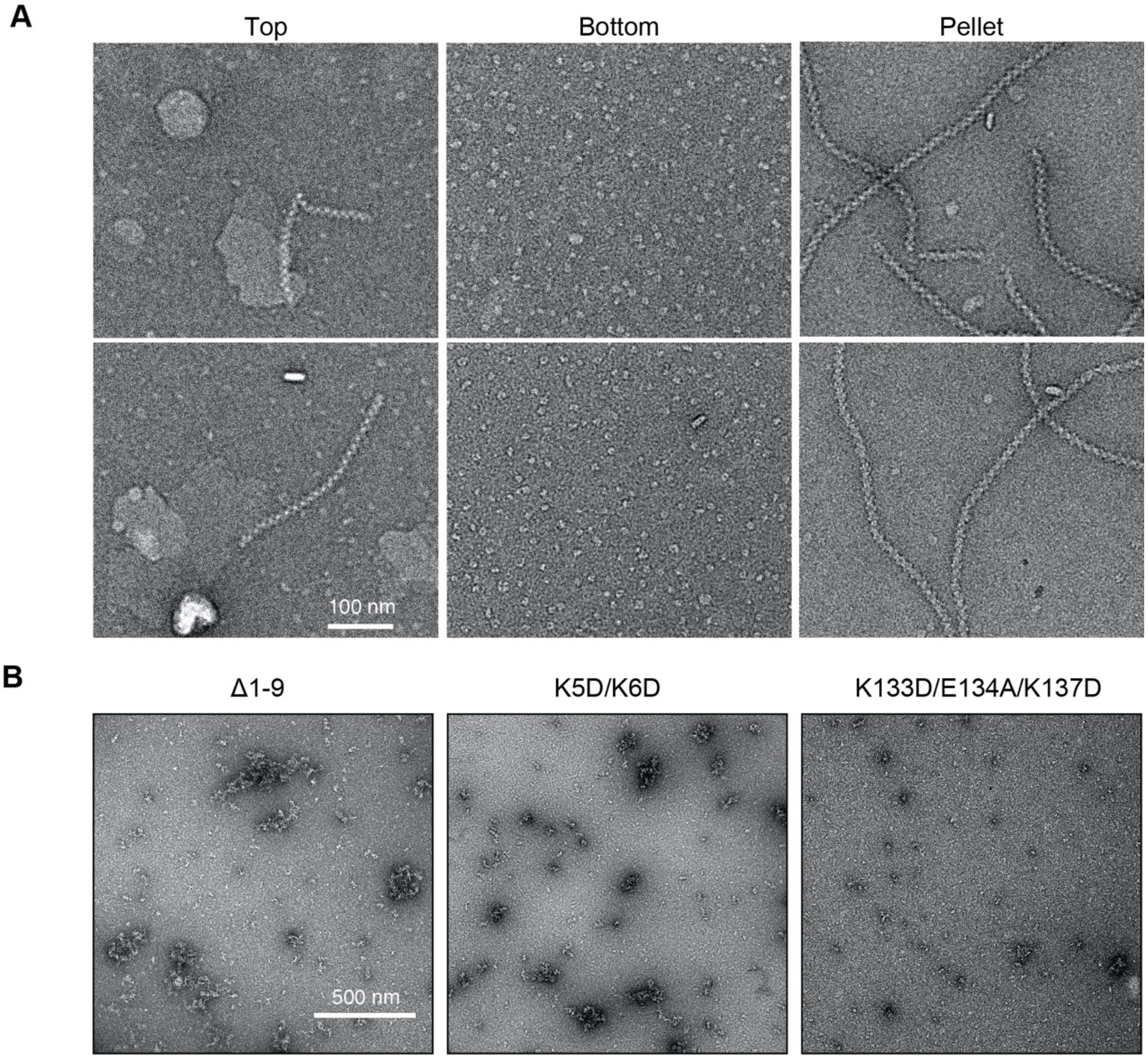

Figure S4: Vps24 liposome flotation assay and Vps24 filament-breaking Vps24 mutants.

(A) Two representative micrographs of the liposome flotation assay for each fraction: top floating (left) contains liposomes and shorter filaments. Bottom (center) does neither contain liposomes nor filaments but smaller oligomers, Pellet (right) shows long filaments and no liposomes. (B) Negative stain electron micrographs of positive residue Vps24 mutants: Δ1-9, K5D/K6D and K133D/E134A/K137D show no filament formation.

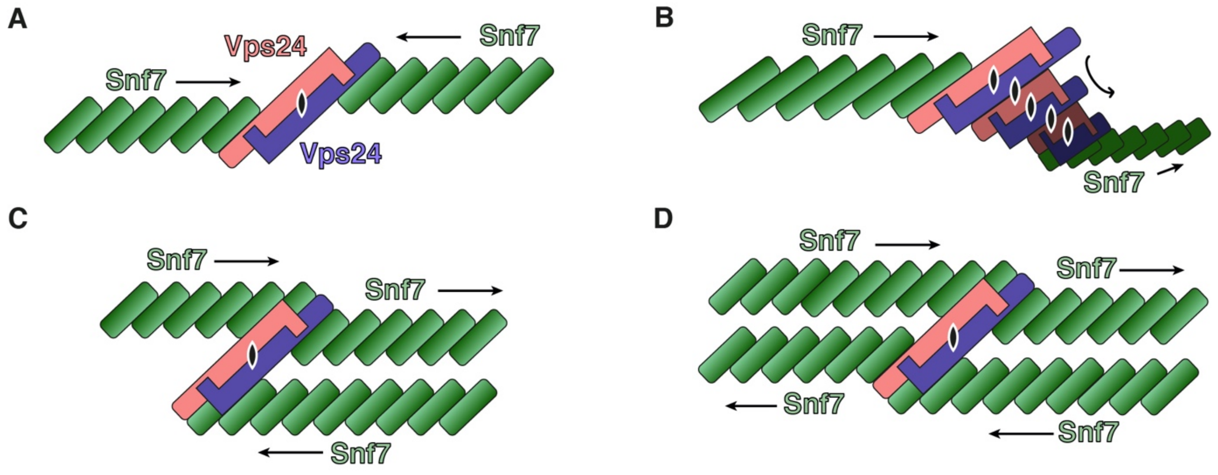

Figure S5: Models of Vps24 geometry modulation in linear Snf7 filaments.

(A) Incorporation of a single Vps24 dimer in a filament of Snf7 monomers can connect Snf7 filaments of opposing polarity. (B) Incorporation of three Vps24 dimers in a linear Snf7 filament of monomers induces a torsional twist from a two-dimensional (flat) polymer to a three-dimensional assembly. (C) A Vps24 dimer could act as an adapter molecule branching one linear Snf7 filament into two filaments with opposing polarities. (D) Vps24 could also connect two linear Snf7 filaments by side-to-side joining.
